# Multi-Drug Resistant Coliform: Water Sanitary Standards and Health Hazards

**DOI:** 10.3389/fphar.2018.00311

**Published:** 2018-06-12

**Authors:** Meerambika Mishra, Ananta P. Arukha, Amiya K. Patel, Niranjan Behera, Tapan K. Mohanta, Dhananjay Yadav

**Affiliations:** ^1^School of Life Sciences, Sambalpur University, Sambalpur, India; ^2^Department of Infectious Diseases and Pathology, University of Florida, Gainesville, FL, United States; ^3^UoN Chair of Oman’s Medicinal Plants and Marine Natural Products, University of Nizwa, Nizwa, Oman; ^4^Department of Medical Biotechnology, Yeungnam University, Gyeongsan, South Korea

**Keywords:** coliform, *E. coli*, fecal pollution, multi-drug resistance, indicator bacteria, gut microflora, MAR index

## Abstract

Water constitutes and sustains life; however, its pollution afflicts its necessity, further worsening its scarcity. Coliform is one of the largest groups of bacteria evident in fecally polluted water, a major public health concern. Coliform thrive as commensals in the gut of warm-blooded animals, and are indefinitely passed through their feces into the environment. They are also called as model organisms as their presence is indicative of the prevalence of other potential pathogens, thus coliform are and unanimously employed as adept indicators of fecal pollution. As only a limited accessible source of fresh water is available on the planet, its contamination severely affects its usability. Coliform densities vary geographically and seasonally which leads to the lack of universally uniform regulatory guidelines regarding water potability often leads to ineffective detection of these model organisms and the misinterpretation of water quality status. Remedial measures such as disinfection, reducing the nutrient concentration or re-population doesn’t hold context in huge lotic ecosystems such as freshwater rivers. There is also an escalating concern regarding the prevalence of multi-drug resistance in coliforms which renders antibiotic therapy incompetent. Antimicrobials are increasingly used in household, clinical, veterinary, animal husbandry and agricultural settings. Sub-optimal concentrations of these antimicrobials are unintentionally but regularly dispensed into the environment through seepages, sewages or runoffs from clinical or agricultural settings substantially adding to the ever-increasing pool of antibiotic resistance genes. When present below their minimum inhibitory concentration (MIC), these antimicrobials trigger the transfer of antibiotic-resistant genes that the coliform readily assimilate and further propagate to pathogens, the severity of which is evidenced by the high Multiple Antibiotic Resistance (MAR) index shown by the bacterial isolates procured from the environmental. This review attempts to assiduously anthologize the use of coliforms as water quality standards, their existent methods of detection and the issue of arising multi-drug resistance in them.

## Introduction

Water, the elixir of life is indispensable in every facet of existence. Apart from quotidian uses, water is also important in many industries for the production of chemicals, cosmetics, and beverages (Lamikanra, 1999; Mishra et al., 2012). The importance of rivers, the major source of freshwater, is well evidenced by the historic fact that major civilizations were settled along the banks of a river. However, water’s indispensability is handicapped by its pollution which renders it unusable. Water polluted by influx of sewage especially from hospitals, public defecation, slaughter houses, clinics, and animal husbandry contains huge amount of antibiotic resistant bacteria which can ultimately be transferred to humans or livestock (Ahmed et al., 2010; Crimmins and Beltrán-Sánchez, 2011; Carlet et al., 2012). One of the major causes of child mortality under five is waterborne illnesses (WHO and UNICEF, 2000; Howard and Bartram, 2003). The group of coliform bacteria from the family *Enterobacteriaceae* was chosen as water potability indicators as they inherently populate the gut of warm blooded mammals; end up in their feces and indicate the presence of other pathogens. (Berg and Metcalf, 1978; Mishra et al., 2012; Mishra et al., 2013). These bacteria are very receptive to drug resistance genes and also readily propagate it to the other pathogens prevalent in the vicinity, therefore pose as potent health hazards. Hence, regular monitoring of coliform levels in the environment provides insight into the status of water potability, warns prior incidence of various public health concerns and paves way for designing remedial measures.

The present review focuses on the concern of fecal pollution in surface waters, the use of coliform as water quality indicators, and the alarming incidence of multi-drug resistance in coliforms posing as health risk.

## Pollution of River Water and Coliform as Sanitary Standards

The United Nations confirms that almost 2 million tons of waste finds its way to the water bodies globally (UNWAP, 2003; Ross, 2010). About 1.2 billion people defecate along the banks of the rivers in developing and under-developed nations (WHO and UNICEF, 2008). Seventy percentage of the industrial wastes have been estimated to be disposed untreated into the water supplies (UN-WATER, 2009). Although the treated sewage allowed to be discharged into the rivers should not exceed the Biochemical Oxygen Demand (BOD) limit of 30 mg/L, Total Suspended Solids (TSS) limit of 50 mg/L (Tyagi et al., 2013), a mere 18% of the 33,000 mld sewage spawned daily is treated. Several investigations have been undertaken over time to monitor water quality and more precisely its potability; among all the parameters available, the microbiological ones largely influence water usability, as has been established by many studies reporting high levels of contamination in the freshwater bodies (WHO, 2006; Mishra et al., 2012, 2013; Harnisz et al., 2015; Parvez et al., 2016; Gomes Freitas et al., 2017).

### Fecal and Non-fecal Coliform

The group of coliform is further subdivided into total and fecal coliforms while total includes both soil intermediates and fecal forms, fecal coliforms confines to those from fecal origin, used as standard microbial indicators of water quality since 1920 (Ashbolt et al., 2001). The fecal coliform group, i.e., *Escherichia coli* (*E. coli*), *Klebsiella*, and *Enterobacter* inhabit the intestinal tract of warm blooded animals (Dufour, 1977). The use of coliform bacteriological analysis for water potability testing dates back to 1880 when Von Fritsch found *Klebsiella pneumoniae* and *K. rhinoscleromatis* in human feces (Geldreich, 1978). Even the USEPA recommends the use of *E. coli* as efficient indicator for evaluating freshwater quality (United States Environmental Protection Agency [USEPA], 1986).

### Methods of Detection- Past and Present

**FIGURE 1:**
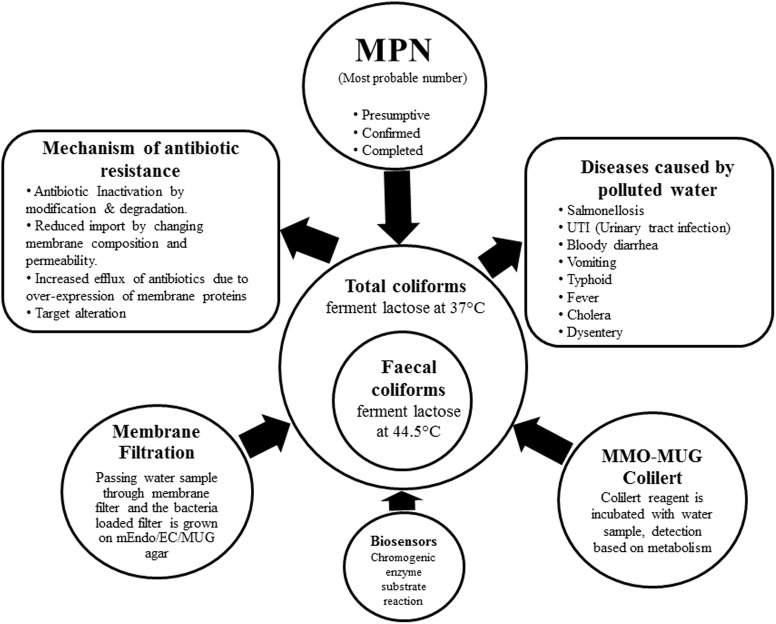
The figure lucidly depicts the categorization of coliforms into total coliforms including both soil intermediates and from faecal origin and strictly faecal coliforms. The different methods of detection of coliform from water sample are enumerated. Mechanism of antibiotic resistance developed by coliform and diseases caused by the use of polluted water have been illustrated in the diagram.

Percy and Grace counted bacteria from water on Robert Koch’s solid gelatin media (Hutchinson and Ridgway, 1977), then Theodor Escherich discovered *Bacillus coli* (Escherich, 1885); later renamed as *E. coli* by Castellani and Chambers (1919). By 1893, the ‘Wurtz method’ of enumerating *B. coli* by directly plating water samples on litmus lactose agar employing the idea of acid formation from lactose fermentation was used by sanitary bacteriologists (Ashbolt et al., 2001). In 1893 Durham’s tubes were used for detecting gas formation from fermentation (Durham, 1898). In 1905 MacConkey’s broth was used as a diagnostic for bile tolerant lactose-fermenting bacteria (MacConkey, 1905). Till date the most preferred and cost-effective method for elaborate detection of coliforms is the Most Probable Number (MPN) comprising of the presumptive, confirmed and completed test. A set of double and single strength lactose broth with inverted durham tubes are inoculated with measured amounts of water to be tested, coliform ferment the lactose present in the broth producing acid and gas. The MPN of coliforms present in 100 ml of water is estimated by the number of positive tubes against the MPN table. Samples from the positive tubes are streaked on EMB agar plates in the confirmed test, discrete black nucleated colonies with green metallic sheen are *E. coli*. Single colonies are re-inoculated into the Brilliant Green Bile Lactose Broth, while bile inhibits the growth of gram-positive microbes; brilliant green bars the propagation of certain gram-negative bacilli allowing the growth of lactose-fermenting coliforms (Mishra et al., 2012, 2013). To test for thermo-tolerant coliform modified Eijkman test has been proposed by the WHO based on the principle that only fecal coliforms are able to ferment lactose at 44.5°C, hence MacConkey broth with inverted durham’s tubes are inoculated with measured amounts of water samples and are incubated at 44.5°C for the occurrence of acid and/or gas (WHO, 2011). Other existent methods of detection are the membrane filter and MMO-MUG (Colilert^®^). The different methods of detection have been illustrated in the **Figure 1** along with categorization of coliform, their health hazards and mechanism of drug resistance.

An efficient sensor based presence/absence method has also been established for confirming the presence of *E. coli.* The chromogenic substrate β-galactosidase enzyme assay detects the color change through a live webcam with Wi-Fi connected sensor node, in the water sample when mixed with X-gal containing reagent (Kim and Myung, 2015). Biosensors have also been developed to check the presence of waterborne pathogens in food (Thakur and Ragavan, 2013). Although several other methods such as ELISA and PCR are also existent, however, they are laboratory based methods which necessitate insolation, pure culture of the organism, also require expert personnel, expensive instruments, multiple steps and do not offer real-time data (Yoon and Kim, 2012). With relatively fast generation times but inability to survive longer in limited water samples the duration from field-to-lab massively affects the coliform numbers often leading to inaccurate quantification. Paper based microfluidic diagnostic devices though relatively inexpensive are largely single test based (Yetisen et al., 2013). Nonetheless, when it comes to large water bodies being polluted mere detection of coliform presence is not enough, it needs complete quantitation of the degree of pollution for remedial steps to be devised; hence inexpensive traditional counting methods which give real-time numbers are preferred. So far only one study has professed the use of sensor for detecting and enumerating *E. coli* in water (Hesari et al., 2016). All the methods available have been enlisted in **Table 1**.

**Table 1:** The various methods developed to detect the presence of coliforms.

Method (Use of specific media/method/chemical)	Principle	References
Use of Robert Koch’s solid gelatin media	Counting bacteria based on colony forming unit	Hutchinson and Ridgway, 1977
Wurtz method	Enumerating *B. Coli* based on lactose fermentation	Ashbolt et al., 2001
Use of durham’s tubes	Positive identification of coliform based on acid and gas production from lactose fermentation	Durham, 1898
Use of MacConkey’s broth	Coliform identification based on being bile tolerant lactose fermenters	MacConkey, 1905
Most Probable Number	Quantitative test recommended by the World Health Organization. Based on lactose fermentation ability of coliforms forming acid and gas, capability of *E. coli* to reduce methylene blue to exhibit green metallic sheen on Eosin Methylene Blue agar, competence of coliforms to ferment lactose producing gas in the presence of bile	American Public Health Association (APHA), 1992; Clesceri et al., 1996 Mishra et al., 2012; Mishra et al., 2013
Modified Eijkman test	Quantitative test recommended by the World Health Organization. Based on lactose fermentation knack of strictly fecal coliforms forming acid and gas	WHO, 2011;Mishra et al., 2012, 2013
Membrane filtration	Usage of filters would trap the coliforms which could be counted in terms of CFU on media	World Health Organization [WHO] (1996)
MMO-MUG (Colilert^®^, Coliscan^®^, and Colitag^®^)	Enzyme based detection of *E. coli*	United States Environmental Protection Agency [USEPA], 2002
ELISA and PCR	Antibody specificity	Yoon and Kim, 2012
Paper based microfluidic diagnostics	Based on catalase test	Yetisen et al., 2013
Biosensors	Chromogenic substrate- enzyme interaction based	Thakur and Ragavan, 2013; Kim and Myung, 2015; Hesari et al., 2016


### Need for Universally Uniform Regulatory Guidelines, Continued Surveillance and Detailed Studies

Monitoring fecal pollution of waters used for recreational drinking, and industrial purposes is imperative for public health and economic reasons. Pathogens introduced from fecal contamination leads to diseases in humans (typhoid, Salmonellosis, cholera), livestock and economic losses to industries (Ballester and Sunyer, 2000; Bernhard and Field, 2000).

Coliform densities are influenced by seasonal changes. While in the tropical climate coliform levels are higher during monsoon, owing to sewage seepage and runoffs, in the comparatively cooler regions they are higher during summer due to ambient growth temperature (Kar et al., 2010; Galadima et al., 2011; Panigrahi and Patra, 2013). This geographically inconsistent season based variation in coliform numbers had led to ambiguous standards which don’t hold context in every reference. The stringency in the guidelines was finalized owing to global health concern reports. As per the WHO, there should not be any *E. coli* or thermo-tolerant coliform detectable in 100 ml of water sample (WHO, 1998, 2011).

Most studies conducted on coliforms only report the MPN level indicating the water potability and no detailed work to further identify and type the procured coliform is done. Routine cultural characterization, biochemical fingerprinting, antibiogram analysis and molecular typing are absolutely indispensable for their genotypic and phenotypic characterization. Even within the same species there occur differences because of variations in the source of bacterial origin, their present habitat and mutations which are unraveled in taxonomic studies.

While coliforms are established as sanitary standards, the startling issue of multi-drug resistance in them is also was on the rise. Coliforms are extremely receptive and altruistic to horizontal gene transfer in nature (Mishra et al., 2013; von Wintersdorff et al., 2016). Hence, many aberrations to the stipulated traits of coliforms have been reported. Unusual Sucrose fermentation was reported by Palchaudhuri et al. (1977) the multi-drug resistant (MDR) strain of *E. coli* also carried genes for resistance against Streptomycin, Ampicillin, Tetracycline, Chloramphenicol, and sulphonamides. Atypical raffinose fermentation linked with Verocytotoxigenic *E. coli* (de Lopez et al., 1982; Saif et al., 2008) is plasmid mediated with genes for antibiotic resistance (Orskov and Orskov, 1973; Cornelis et al., 1978). Non-fermentation of rhamnose is a decisive factor of VT+*E. coli* 0157 (Chapman et al., 1991). The biotype (Rha- and Suc-)” is a quintessential marker for the preliminary detection of SLT-I+ *E. coli* in SLTEC associated diarrhea (Wieler et al., 1995). The occurrence of certain atypical carbohydrate fermentation genes is largely coupled with multi-drug resistance. These genes are transferred both intra and inter-specifically through horizontal gene transfer mechanism bestowing multi-drug resistance to the isolates which changes their biochemical characteristics. Numerous investigations employ biochemical fingerprinting for identifying coliform (Gililland and Vaughn, 1943; Kühn et al., 1991; Guentzel, 1996; Betsy and Keogh, 2005). While results obtained by most of the biochemical tests are qualitative and used mostly for the identification of the bacterial isolate into genus and species but carbohydrate fermentation, antimicrobial susceptibility, genotypic profiling characterize the bacterial isolate down to the strain level. When coupled together these analyses well substantiate the identification of an individual strain with detailed characterization of its standalone traits. Carson et al. (2001) have used 16S rDNA analysis to discriminate fecal *E. coli* of human source from collective fecal *E. coli* isolates of non-human origins and have obtained accuracy up to three host sources. 16S rDNA analysis has been used to differentiate isolates within the same species based on taxonomic classification (Laurent et al., 1996), epidemiological tracking (Bernhard and Field, 2000; Zhao et al., 2005), geographical distribution (Akhtar et al., 2000), population biology and phylogeny (Paradis et al., 2005; Xia et al., 2013).

## Antibiotic Resistance in Gut Coliform

Though tagged as “miracle drugs,” antibiotics are increasingly being misused. Their unsystematic use and dumping has led to multi-drug resistance among the indigenous microbiota, in case of a re-infection these antibiotics become futile for therapy. The increasing use of antibiotics in clinical, agricultural and veterinary sectors is analogous with the escalated resistance of bacteria to these frequently used antibiotics (Dhanorkar and Tambekar, 2004; Ahmed et al., 2010). This has exaggerated the expenditure of therapy, the peril of spreading MDR, mortality from diseases through the MDR strains (Martínez and Baquero, 2002; White, 2006; Hawkey and Jones, 2009; Kolar et al., 2010). Antibiotic resistance has been established to be broadcasted in the gut of the warm-blooded animals (Chopra and Roberts, 2001; Launay et al., 2004; Fukao and Yajima, 2012). Studies by Larsson and Fick (2009) and Laxminarayan and Heymann (2012) have reported seepage of about 45 kg of Ciprofloxacin quotidian from factories into the nearby water bodies. Spread of environmental pollution has led to the unearthing of antibiotic-resistant bacteria in uninhabited lands like Antarctica (Sjölund et al., 2008; Hernández et al., 2012). Prolongation of disease either due to scarcity of antibiotics or superfluous use of antibiotics in circumstances where their use is not obligatory is common. Antibiotics usage has seen a precipitous boost over the past few years. Sale of cephalosporins amplified by 60% over 2005–2009, sales of other last line antibiotics escalated six-fold from 2005 to 2010 (Ganguly et al., 2011; Westly, 2012). Drug resistant coliform are the most commonly infecting bacteria, infection is contracted by exposure to unhygienic food, water, to the bacteria or its carriers and unwise use of antibiotics. Hannah et al. (2005) have investigated the association of drug resistant *E. coli* infections with epidemiological factors: demographic variables, diet, healthcare and antibiotic usage and have found positive correlation between infection with MDR *E. coli* and contact with infected persons, contaminated food, ambulatory visit, antimicrobial misuse, prolonged medication and frequent meat consumption with self-prescription of drugs. Over-consumption of antibiotics kills the beneficial bacteria in our system enhancing the growth of pathogens which may be insensitive to the antibiotics used thus eventually becoming resistant. Consuming antibiotics lesser than the prescribed amount does not kill the pathogen rather confers resistance upon it. In both the cases treating the MDR bacteria becomes arduous with often, fatal consequences. Antimicrobial susceptibility with the production of Extended Spectrum Beta-Lactamases (ESBL) among the coliform isolates causes concern as they render therapy with beta-lactum antibiotics inefficient (Pitout and Laupland, 2008; Peirano and Pitout, 2010; Rogers et al., 2011; Shibl et al., 2012).

Many investigations have stressed on the concern of multi-drug resistant coliforms in water (Manji et al., 2012; Mishra et al., 2013; Al-Agamy et al., 2014; El-Zanfaly, 2015; Madec et al., 2016; Azzam et al., 2017; Jutkina et al., 2018). Manji et al. (2012) have emphasized concern over the prevalence of multi-drug resistant coliforms and *Staphylococcus aureus*, in rural water supplies of Nigeria. Over the period of several months their analysis involved bacteria counting, antibiogram and MIC analysis. The presence of pathogens as *S. aureus*, *Bacillus sp.*, *Pseudomonas aeruginosa*, with umpteen antibiotics resistant *E. coli* strains warrant immediate treatment as they pose momentous public health repercussions.

Mishra et al. (2013) have worked on the prevalence of antibiotic resistant *E. coli* in the River Mahanadi. The water there unintentionally experiences influx of miscellaneous composition. They have employed MPN, Modified Eijkman test, biochemical fingerprinting, antimicrobial susceptibility testing and 16S rDNA ribotyping methods to isolate, enumerate and identify the coliform isolates to be *E. coli* with established resistance to beta-lactum antibiotics, carboxypenicillin coupled with β-lactamase inhibitor, glycopeptides, carbapenems, macrolides, till fourth generation of fluoroquinolones cephalosporins. They have also reported appalling resistance rates of the isolates against 42 indigenously used antibiotics with MAR indices ranging from 0.51 to 0.90 which indicate severe pollution and risk to the public health.

Transfer of antibiotic resistance cannot be ascribed to horizontal gene transfer mechanisms alone rather integrons too play an important role in certain cases. Xia et al. (2013) used 16S rDNA analysis to accurately isolate coliforms, harboring class 2 integrons and investigated their molecular architecture.

Al-Agamy et al. (2014) have characterized the prevalence of ESBL *Escherichia coli* (ESBL-EC) in Riyadh. They used antimicrobial susceptibility and pulsed field gel electrophoresis (PFGE) to type and investigate the epidemiology of ESBL-EC and the prevalence of ST131 in them. They have also expressed concern over the dearth of local reports on the prevalence of ESBL-EC despite its universal predominance. Such state of affairs demands the routine and vigilant inspection of the environment.

El-Zanfaly (2015), apprehends that resistance to antibiotics may actually escalate during the sewage treatment processes his study has employed enumeration, antimicrobial susceptibility, granular activated carbon test and transferability of antibiotic resistant character tests to characterize ampicillin, sulfaguanidine penicillin, 2-sulfanilamide pyrimidine, tetracycline, chloramphenicol, neomycin and streptomycin resistant coliforms. Activated carbon application in pilot water treatment plant experiment showed their prevalence with easy transferability of resistance. The work insists on including antibiotic resistance in coliforms as a deciding criterion for judging water quality standards.

Madec et al. (2016) have stressed on the implication of antimicrobial resistance in drinking water as a potent risk indicator for humans in low-income nations. A particular *E. coli* isolate ST48, procured from drinking water in France, resistant to ceftiofur, tetracyclines and sulfonamides was found to harbor the blaCTX-M-1 IncI1/ST3 plasmid. The plasmid is analogous to the other blaCTX-M-1 IncI1/ST3 plasmids that have been reported in other animals and humans. Based upon their findings they suggest the possibility of human transfer of ESBL genes through drinking water in developing and under-developed nations.

Jutkina et al. (2018) conducted a series of conjugation and MIC assays of both donor and recipient strains to investigate the latent of cefotaxime, ciprofloxacin, gentamicin, erythromycin, sulfamethoxazole, trimethoprim, and chlorhexidine digluconate, hexadecyl trimethyl ammonium chloride and triclosan to stimulate horizontal gene transfer of antibiotic resistance genes. They found that 24.4 μg/L Chlorhexidine (200 times <MIC of the recipient) 0.1 mg/L triclosan (1/20 < MIC), 0.1 mg/L gentamicin (1/16 < MIC) and 1 mg/L sulfamethoxazole (1/16 < MIC) potently escalated the frequencies of antibiotic resistance gene transfer. The study clearly establishes that the presence of certain antimicrobials below their MICs in nature can induce the horizontal transfer and spread of antibiotic resistance.

## Conclusion

It is indispensable to study the bacterial isolates endemic to an ecological area in order to devise preventive and remedial mechanisms in the event of nuisance created by it. Owing to the exorbitant gene transfer mechanisms bacteria have been found to vary epidemiologically even within the same species. Knowledge of clinically imperative attributes as virulence and antimicrobial susceptibility is absolutely indispensable both for designing remedial measures and preventive therapy. Therefore, it is essential to thoroughly characterize and report different isolates that vary and adapt according to the changing surroundings. The work holds utmost importance in developing and under-developed countries where parameters for regular monitoring of water quality is limited and advanced molecular biology tools for immediate identification are not ubiquitous, field-to-lab distance is more, availability of skilled personnel is scarce, seasonal abundance of coliform is atypical and quotidian use of natural water body is prevalent. Thus, isolation and complete characterization of water-borne bacteria are quintessential to answer clinically relevant problems.

## Author Contributions

MM designed, conceived, and wrote the manuscript. AA and AP helped in writing. NB, TM, and DY critically reviewed, edited, and finalized the manuscript for submission.

## Conflict of Interest Statement

The authors declare that the research was conducted in the absence of any commercial or financial relationships that could be construed as a potential conflict of interest.
